# Assessing the Impact of Excessive Gestational Weight Gain Among Women With Type 1 Diabetes on Overweight/Obesity in Their Adolescent and Young Adult Offspring: A Pilot Study

**DOI:** 10.3389/fendo.2018.00713

**Published:** 2018-11-30

**Authors:** Ketrell L. McWhorter, Katherine Bowers, Lawrence Dolan, Ranjan Deka, Chandra L. Jackson, Jane C. Khoury

**Affiliations:** ^1^Division of Biostatistics and Epidemiology, Cincinnati Children's Hospital Medical Center, Cincinnati, OH, United States; ^2^Department of Environmental Health, University of Cincinnati, College of Medicine, Cincinnati, OH, United States; ^3^Epidemiology Branch, National Institute of Environmental Health Sciences, National Institutes of Health, Department of Health and Human Services, Research Triangle Park, NC, United States; ^4^Department of Pediatrics, University of Cincinnati, College of Medicine, Cincinnati, OH, United States; ^5^Division of Endocrinology, Cincinnati Children's Hospital Medical Center, Cincinnati, OH, United States

**Keywords:** Type 1 diabetes mellitus, gestational weight gain, overweight, obesity, offspring

## Abstract

**Aims/hypothesis:** We sought to determine the impact of intrauterine exposure to excessive gestational weight gain (GWG) on overweight/obesity in adolescent/young adult offspring of women with type 1 diabetes mellitus (TIDM).

**Methods:** In 2008, a pilot study was conducted among 19 randomly-selected adolescent and adult offspring of mothers with TIDM who participated in the Diabetes in Pregnancy Program Project (DiP) between 1978 and 1995. Body mass index (BMI)-specific Institute of Medicine (IOM) guidelines for gestational weight gain (GWG) were defined as: 12.5–18.0 kilograms (kg) GWG; 11.5–16.0 kg GWG: 7.0–11.5 kg GWG; 5.0–9.0 kg GWG, for women classed as underweight, normal, overweight and obese according to pre-pregnancy BMI, respectively. Generalized estimating equations were used to estimate adjusted odds ratios (aOR, [95% confidence intervals, CI]) for overweight/obesity among offspring, related to IOM adherence, adjusting for pre-pregnancy BMI and mean maternal daily insulin units/kg body weight.

**Results:** Mean age of offspring at follow-up was 20.3 ± 3.3 years, 12(63%) were male, 4(21%) Black and 12(63%) overweight/obese. There were 9(82%) overweight/obese offspring among the 11 mothers who exceeded IOM guidelines for GWG compared with 3(38%) overweight/obese offspring among the 8 mothers with GWG within guidelines. Exceeding vs. adhering to IOM guidelines (OR = 7.50, [95%CI: 0.92–61.0]) and GWG per kilogram (OR = 1.39, [95%CI: 0.98–1.97]) were associated with offspring overweight/obesity at follow-up.

**Conclusions/interpretation:** Our pilot study suggests potential long-term implications of excessive GWG on metabolic health in offspring of mothers with TIDM, warranting future research examining the health impact of GWG in this population.

## Introduction

Perturbations in the fetal environment, such as excessive gestational weight gain (GWG) among women with type 1 diabetes mellitus (TIDM), may increase the offspring's risk of becoming overweight or obese (1). Women with TIDM are at higher risk of obstetric and perinatal complications, such as cesarean section, perinatal mortality and major malformations, compared to those without diabetes (2). Fetuses exposed to both maternal overnutrition and a hyperglycemic intrauterine environment tend to have higher insulin secretion compared with fetuses without these exposures, which may lead to alterations in metabolic programming (3, 4). The resulting state of fetal hyperinsulinemia is hypothesized to be involved in the development of overweight and obesity in offspring (1). Few studies have serially collected clinical maternal measures during pregnancy that allow for the longitudinal examination of time-specific exposure to GWG in women with TIDM as well as the long-term metabolic outcomes in their young adult offspring (5). Therefore, our aim was to investigate the impact of GWG, pre-pregnancy body mass index (BMI) and maternal insulin requirements over the gestational period on the body mass index (BMI) of offspring at approximately 20 years after birth.

## Materials and methods

A pilot study was conducted in 2008 to investigate the feasibility of a larger follow-up study on health outcomes among the offspring of women who participated in the Diabetes in Pregnancy Program Project (DiP), a randomized clinical trial between 1978 and 1995. There were 336 singleton pregnancies complicated by T1DM, surviving beyond 28 days of life with no known infant deaths, over the course of the study. TIDM in the mothers had been identified in the DiP according to physician confirmation of ketoacidosis, and or c-peptide levels. To identify potential participants for the pilot study, a random list of these aforementioned pregnancies followed in the DiP was created. Contact was attempted for the first 44 on the list, in groups of 8, 32 were located, however one was deceased. Women were asked if a clinic visit could be scheduled for their offspring (if <18 years old) or for permission to directly contact their offspring (if ≥18 years) in order to schedule a clinic visit. Of the 31, 5 refused and 20 were scheduled for a clinic visit, 19 of whom participated. Lack of funding prevented any further recruitment. During this visit each study subject completed questionnaires regarding medical history, health status, diet and physical activity. Institute of Medicine (IOM) guideline adherence for GWG was the primary exposure of interest. GWG was defined as the difference between weight at delivery and self-reported pre-pregnancy weight, GWG adherence to 2009 IOM guidelines was based on specific body mass index (BMI) categories for maternal pre-pregnancy BMI. Women considered within IOM guidelines for GWG consisted of: (1) underweight [BMI- <18.5 kg/m^2^]: 12.5–18.0 kg GWG; (2) normal weight [BMI-≥18.5– <25 kg/m^2^]: 11.5–16.0 kg GWG; (3) overweight [BMI- ≤ 25- < 30 kg/m^2^]: 7.0–11.5 kg GWG; 4) obese [BMI-≥30 kg/m^2^]: 5.0–9.0 kg GWG. Due to sample size and study objective, GWG under (below lower limit of recommended weight gain for the specific BMI categories) and within guidelines were combined and compared to those over guidelines (exceeding upper limit of recommended weight gain for the specific BMI category). The primary outcome of interest was overweight/obese in the offspring at follow-up. Pre-pregnancy BMI in the woman and overweight/obese in the offspring at follow-up was categorized according to BMI as defined above. Maternal glycemic control was characterized using three different measures: mean glycohemoglobin A_1_ [HbA_1_, (%)] per trimester, mean serum pre-prandial and mean 90-minute post-prandial glucose concentrations (mg/dL) collected at clinic visits (6). Concentrations of HbA_1_ were measured at study entry and every 4 weeks using Isolab column chromatography [interassay coefficient of variation 7.2%; normal range (5.5–8.5%)] (7). Insulin per kilogram (units/kg), which included insulin taken per day by injection or base pump and bolus, was calculated using the weight obtained at the same clinic visit. Institutional review board (IRB) approval was obtained from both Cincinnati Children's Hospital Medical Center and the University of Cincinnati.

### Statistical analysis

Under and within IOM guidelines and offspring overweight and obesity were combined for statistical analyses due to the small sample sizes in each category for this pilot study. Continuous variables are represented with mean ± standard deviation (SD) or least squares mean ± standard error of the mean and categorical variables as n (%). Fisher's exact test was used to compare normal and overweight/obese groups for maternal baseline characteristics and offspring outcomes for the categorical variables, due to sample size. *T*-tests were used for normally—and Kruskall–Wallis for non-normally-distributed continuous variables.

Inherent correlation between repeat pregnancies was accounted for by using Generalized Estimating Equations (GEE) when examining odds ratios for exposure to maternal adherence to IOM guidelines and overall maternal weight gain and offspring overweight/obesity.

A *p* < 0.05 was considered statistically significant. Statistical analyses were completed using SAS® software version 9.4 (SAS Institute, Cary NC).

## Results

Our study included 15 mothers and 19 offspring, with 4 mothers having two offspring in the study. Mean ± SD maternal age at delivery was 26.5 ± 4.1 years and 5 (26%) entered pregnancy as overweight/obese (Table 1). In 11 (58%) pregnancies, mothers of the 19 offspring exceeded IOM guidelines for GWG during pregnancy. Mean insulin dose over gestation was higher for mothers who exceeded IOM guidelines (90.1 ± 44.6 mg/dL) compared to mothers under or within guidelines (69.6 ± 23.2, mg/dL), *p* = 0.25.

**Table 1:** Maternal characteristics during pregnancy of women with type 1 diabetes in the DiP (1978–1995) and offspring characteristics at follow-up (2009) overall and by maternal adherence to GWG IOM guidelines, *N* = 19.

	**GWG under/within IOM guidelines^a^**	**GWG over IOM guidelines^a^**		**All**
**Maternal characteristics**	8 (42)	11 (58)	*p*	*n* = 19
Age at delivery (y)	27.6 ± 3.5	25.6 ± 4.5	0.32	26.5 ± 4.1
Married	8 (100)	6 (55)	0.04	14 (74)
Black	1 (13)	3 (27)	0.44	4 (21)
Primaparous	4 (50)	6 (55)	0.84	10 (53)
Pre-pregnancy BMI (kg/m^2^)	21.7 ± 1.9	24.7 ± 5.1	0.10	23.4 ± 4.2
Pre-pregnancy BMI classification			0.08
Normal (18.5 kg/m^2^ to < 25 kg/m^2^)	8 (100)	6 (55)		14 (74)
Overweight (≥25 kg/m^2^ to < 30 kg/m^2^)	0 (0.0)	4 (36)		4 (21)
Obese (≥30 kg/m^2^)	0 (0.0)	1 (9)		1 (5)
Gestational Weight Gain (kilogram) during 1st 20 weeks	4.3 ± 1.7	8.3 ± 4.4	0.04	6.4 ± 3.9
Gestational Weight Gain (kilogram)	12.5 ± 2.4	19.1 ± 3.9	0.0005	16.3 ± 4.6
Preeclampsia	1 (13)	2 (14)	0.74	3 (16)
Previous c-section	2 (25)	2 (14)	0.72	4 (21)
Cesarean section	6 (75)	6 (55)	0.36	12 (63)
Pre-term delivery (prior to 37 weeks' gestation)	2 (25)	2 (18)	0.72	4 (21)
**Maternal diabetes and glucose management**			
Age at diagnosis Insulin-Dependent Diabetes (years)	15.9 ± 6.7	15.8 ± 5.0	0.98	15.8 ± 5.6
White classification			0.37
B	3 (38)	3 (27)		6 (32)
C	1 (13)	5 (45)		6 (32)
D	3 (38)	1 (9)		4 (21)
R (Retinopathy)	1 (13)	2 (18)		1 (5)
RF (Retinopathy and Nephropathy)	1 (13)	1 (9)		2 (11)
Mean Glycohemoglobin A_1_ (HbA_1_) (%)			
First trimester HbA_1_	9.3 ± 1.6	10.5 ± 3.3	0.41	10.0 ± 2.7
Second trimester HbA_1_	7.9 ± 1.0	8.2 ± 1.8	0.71	8.1 ± 1.4
Third trimester HbA_1_	7.3 ± 0.8	7.8 ± 1.6	0.41	7.6 ± 1.3
Mean pre-prandial glucose over gestation^b^	124.3 ± 54.8	135.2 ± 48.9	0.68	131.4 ± 49.7
Mean post-prandial glucose over gestation^b^	156.5 ± 48.0	173.1 ± 32.2	0.38	166.1 ± 39.3
Mean insulin dose over gestation^c^	69.6 ± 23.2	90.1 ± 44.6	0.25	81.5 ± 37.7
Mean insulin dose per kilogram weight over gestation^d^	1.1 ± 0.4	1.1 ± 0.5	0.56	1.1 ± 0.43
Mean Arterial Pressure (MAP) 1st 20 weeks of gestation	84.2 ± 11.9	85.9 ± 7.4	0.74	85.1 ± 9.6
**Offspring characteristics**			
Age of offspring at follow-up (years)	20.8 ± 3.3	19.9 ± 3.4	0.54	20.3 ± 3.3
Male	5 (63)	7 (64)	0.96	12 (63)
Race			
Black	1 (13)	3 (27)	0.44	4 (21)
Birthweight (grams)	3,737 ± 916	3,854 ± 508	0.72	3,805 ± 688
Large for gestational age^e^	5 (63)	7 (64)	0.96	12 (63)
Gestational age at delivery (weeks)	38.0 ± 1.5	38.6 ± 1.7	0.66	38.3 ± 1.6
BMI (kg/m^2^)	24.0 ± 2.7	30.1 ± 8.6	0.39	27.5 ± 7.3
BMI classification			0.07
Normal (18.5 kg/m^2^ to < 25 kg/m^2^)	5 (63)	2 (18)		7 (37)
Overweight (≥25 kg/m^2^ to < 30 kg/m^2^)	3 (37)	5 (45)		8 (42)
Obese (≥30 kg/m^2^)	0 (0)	4 (36)		4 (21)
Offspring waist circumference (cm) over NHLBI guidelines^f^	1 (13)	5 (45)	0.13	6 (32)
Paternal history of diabetes	1 (13)	2 (18)	0.45	3 (16)
Hypertension^g^	1 (13)	0 (0)	0.42	1 (5)

a *BMI-specific 2009 IOM guidelines for adherence were defined as: underweight by BMI: 12.5–18.0 kg GWG; normal by BMI: 11.5–16.0 kg; overweight by BMI: 7.0–11.5 kg GWG; obese by BMI (all classes): 5.0–9.0 kg GWG*.

b *Means were calculated from measurements taken in each trimester*.

c *Mean insulin dose over gestation was calculated from measurements of insulin dose taken at approximate weekly visits over full gestation, including at entry to delivery*.

d *Mean insulin dose per kilogram weight over gestation was calculated by dividing insulin dose taken at approximate weekly measurements over full gestation by participants' weight (in kg) at that visit*.

e *Large for gestational age was defined as infants with a birthweight >90th percentile, according to gestational age, sex and race*.

f *Waist circumference measurements for offspring >88 cm for females and >102 cm for males was defined as exceeding guidelines per NHLBI*.

g *Hypertension was defined as systolic blood pressure ≥130 mm Hg, diastolic blood pressure ≥ 80 mm Hg or physician-prescribed antihypertensive meds*.

*GWG,gestational weight gain; IOM,Institute of Medicine; BMI,body mass index; cm,centimeter; NHLBI,National Heart, Lung and Blood Institute*.

*Missing values: marital status, n = 1 (5); gestational weight gain in 1st 20 weeks, n = 4 (21); mean HbA1 in 1st trimester, n = 5 (26%); mean HbA1 in 2nd trimester, n = 2 (11%); mean preprandial glucose over gestation, n = 2 (11%); mean MAP, n = 3 (16%)*.

*Data are expressed as mean ± standard deviation for continuous variables and n (%) for categorical variables*.

Among the 19 offspring, mean age at follow-up was 20.3 ± 3.3 years and 12 (63%) were male (Table 1). There were 7 (37%) normal weight offspring, and 12 (63%) overweight/obese offspring at follow-up. All mothers of offspring who were normal weight at follow-up were themselves normal weight upon entering pregnancy, while for offspring who were overweight/obese at follow-up 58% of mothers were normal weight upon entering pregnancy (Supplementary Table 1). Mean maternal HbA_1_ of overweight/obese offspring was significantly higher than maternal HbA_1_ of normal weight offspring at the 1st (*p* = 0.01), 2nd (*p* = 0.04), and 3rd (*p* = 0.02) trimesters (Supplementary Table 1).

Exposure to the intrauterine environment of mothers who exceeded IOM guidelines for GWG compared to exposure to the intrauterine environment of mothers who adhered to guidelines conferred a non-significant but over 7-fold increased odds (OR = 7.50, [95%CI: 0.92–61.0], *p* = 0.06) of overweight/obesity at follow-up among offspring (Table 2). After adjusting for pre-pregnancy BMI the association was slightly attenuated (aOR = 4.49, [95%CI: 0.45–44.5], *p* = 0.20).

**Table 2:** Odds Ratios (OR) and adjusted Odds Ratios (aOR) for overweight/obesity among offspring at follow-up of women with type 1 diabetes mellitus in the DiP (1978-1995), using separate models of Gestational Weight Gain Adherence and Total Gestational Weight Gain.

	**Model I**		**Model II**		**Model III**
***N* = 19**	**OR (95% CI)**	***p***	**aOR (95% CI)**	***p***	**aOR (95% CI)**	***p***
IOM adherence^a^					
Under/Within IOM guidelines	Reference		Reference		Reference
Over IOM guidelines	7.50 (0.92-61.0)	0.06	4.49 (0.45-44.5)	0.20	Not estimable
Total gestational weight gain (kilograms)^b^	1.39 (0.98–1.97)	0.07	1.25 (0.86–1.81)	0.23	1.51 (0.96–2.39)	0.07

Model I—unadjusted

Model II—Model I and pre-pregnancy BMI (kg/m^2^) as continuous

Model III—Model I and mean insulin (mg/dL) dose per kilogram weight over total gestation

a BMI-specific 2009 IOM guidelines for adherence were defined as: underweight by BMI: 12.5–18.0 kg GWG; normal by BMI: 11.5–16.0 kg; overweight by BMI: 7.0–11.5 kg GWG; obese by BMI (all classes): 5.0-9.0 kg GWG

b *Total gestational weight gain (kg) was calculated as (weight at delivery)—(weight at LMP)*.

*Participant's mean insulin dose per kilogram weight over gestation was calculated by dividing insulin dose (mg/dL) taken at approximate weekly measurements over full gestation by participant's weight (in kg) at that visit*.

*CI, 95% confidence interval; IOM, Institute of Medicine; BMI, body mass index (kg/m^2^)*.

Examining the actual weight over pregnancy rather than adherence to IOM guidelines showed a similar association. The unadjusted odds for exposure to increasing GWG (per kg) and overweight/obesity among offspring at follow-up was OR = 1.39, [95%CI: 0.98–1.97], *p* = 0.07 (Table 2). After adjusting for pre-pregnancy BMI, the relationship between total GWG (per kg) and offspring BMI was slightly attenuated (aOR = 1.25, [95%CI: 0.86–1.81], *p* = 0.23). Adjusting for mean insulin dose per maternal body weight (kg) over gestation, total GWG per kg was associated with non-statistically significant increased odds (aOR = 1.51, [95%CI: 0.96–2.39], *p* = 0.07) for overweight/obesity among offspring at follow-up.

## Discussion

In this pilot study of 19 offspring, we identified a high prevalence of mothers exceeding IOM guidelines for GWG, in addition, these mothers were more likely to have overweight/obese offspring at follow-up compared to mothers staying under/within IOM guidelines. Nine of the 12 overweight/obese offspring had mothers who exceeded IOM guidelines. All five mothers who entered pregnancy as overweight/obese according to BMI had overweight/obese offspring at follow-up, while all offspring who were normal weight had mothers who entered pregnancy with a normal BMI. Even with a small sample size, we observed an association between mothers exceeding IOM guidelines for GWG and overweight/obesity outcomes among young adult offspring in this population, albeit with borderline statistical significance. Prior studies have established associations between excessive GWG and LGA at birth (8, 9). In addition, there is an increased risk of adult overweight and glucose intolerance among offspring of women with diabetes who are born overweight (5). Secher et al. found a positive association between higher GWG and increased offspring birth weight, independent of glycemic control and prepregnancy BMI (9). In our study, nearly 60% of offspring who were overweight/obese at follow-up were born LGA, while only 42% of normal weight offspring were born LGA. Although we did not adjust for LGA or maternal glucose control, we observed a slight increase in the odds of overweight/obesity among offspring with exposure to maternal weight gain per kg after adjusting for maternal insulin per kg during pregnancy. However, given the observed significant differences in glucose control throughout gestation among mothers of normal weight offspring compared to overweight/obese offspring, our results suggest important clinical implications if, indeed, we are observing a true association. Exposure to excessive GWG, reported by some as particularly important in early pregnancy, (10) during which time metabolic programming of the fetus begins, may lead to long-term effects on the metabolic health of offspring (3, 4, 5). Appropriate GWG and glucose management is paramount for this population, particularly for women entering pregnancy as overweight/obese. As our results show these women are also more likely to have higher HbA_1_ throughout gestation, exceed IOM guidelines for total GWG and have higher GWG during early pregnancy.

Limitations of our study include the small sample size, restricting our ability to perform statistical analysis to examine fully adjusted associations and possible interactions. Although data were available, it was also difficult to account for potential modifiers, including maternal and offspring smoking status, breast feeding as an infant, paternal history of diabetes, offspring dietary intake, and reduced offspring physical activity. Although our sample included only four black offspring, this proportion is representative of the T1DM population and this was a randomly selected group. Despite the limitations, an important strength of this study was in the comprehensive, longitudinal data from the original cohort, with repeated clinical measures over pregnancy and comprehensive follow-up data on offspring. This follow-up study aimed to demonstrate feasibility of a larger study and successful recruitment of adult offspring of women in the DiP cohort.

Our pilot study suggests that there are long-term implications of increasing maternal GWG on overweight/obese outcomes in offspring of mothers with TIDM. Our data also demonstrate the importance of identifying modifiable behaviors, including maternal adherence to IOM guidelines for GWG and adequate glucose control throughout pregnancy, to reduce metabolic adversities in adult offspring. Future directions include following up on a larger number of participants in this cohort to evaluate the long-term impact of exposure to the intrauterine TIDM environment.

## Data sharing statement

The datasets for this manuscript are not publicly available because there is an ongoing funded R01 for follow-up of 250 offspring. Requests to access the datasets should be directed to JK (jane.khoury@cchmc.org).

## Author contributions

KM and JK study concept and design; JK acquisition of data; KM statistical analysis; KM, KB, LD, RD, CJ, and JK interpretation of data; KM drafting of the manuscript; KM, KB, LD, RD, CJ, and JK critical revision of the manuscript for important intellectual content; CJ and JK administrative, technical, and material support.

### Conflict of interest statement

The authors declare that the research was conducted in the absence of any commercial or financial relationships that could be construed as a potential conflict of interest.
