# Supplementary material for: Assessing the Impact of Excessive Gestational Weight Gain Among Women With Type 1 Diabetes on Overweight/Obesity in Their Adolescent and Young Adult Offspring: A Pilot Study
Source: Front Endocrinol (Lausanne). 2018 Nov 30;9:713. doi: 10.3389/fendo.2018.00713 (PMC6287600; doi:10.3389/fendo.2018.00713)
Supplement: Supplementary file 1 [file Table_1.pdf]

# Assessing the Impact of Excessive Gestational Weight Gain among Women with Type 1 Diabetes and Overweight/Obesity in their Adolescent and Young Adult Offspring: A Pilot Study

Ketrell L. McWhorter PhD, MBA[1,2,3], Katherine Bowers PhD [1,2,4], Lawrence Dolan MD[4,5], Ranjan Deka PhD [2], Chandra L. Jackson PhD, MS [3], Jane C. Khoury PhD [1,2,4,5]

[1]Division of Biostatistics and Epidemiology, Cincinnati Children's Hospital Medical Center, Cincinnati, OH, USA

[2] Department of Environmental Health, University of Cincinnati, College of Medicine, Cincinnati, OH, USA

[3] Epidemiology Branch, National Institute of Environmental Health Sciences, National Institutes of Health, Department of Health and Human Services, Research Triangle Park, NC, USA

[4] Department of Pediatrics, University of Cincinnati, College of Medicine, Cincinnati, OH, USA

[5] Division of Endocrinology, Cincinnati Children's Hospital Medical Center, Cincinnati, OH, USA

| | |
|---|---|
| Keywords: | Type 1 Diabetes Mellitus, Gestational Weight Gain, Overweight, Obesity, Offspring |
| Running Title: | Overweight/Obesity in offspring of mothers with TIDM |
| Date: | June 14, 2018 |

**Corresponding Author:**
Jane C. Khoury, PhD
Jane.khoury@cchmc.org

**Supplementary Table 1. Maternal characteristics during pregnancy of women with type 1 diabetes in the DiP (1978-1995) and offspring characteristics at follow-up (2009) by offspring BMI classification, N=19**

| | Normal weight | Overweight / Obese | | All |
|---|---|---|---|---|
| | 7 (37) | 12 (63) | *p* | 19 |
| Age at delivery (y) | 28.6±3.7 | 25.3±4.0 | 0.09 | 26.5±4.1 |
| Married | 6 (86) | 8(67) | 0.11 | 14 (74) |
| Black | 0 (0) | 4 (33) | | 4 (21) |
| Primaparous | 4 (57) | 6 (50) | 0.76 | 10 (53) |
| Pre-pregnancy BMI (kg/m$^2$) | 21.3±2.0 | 24.6±4.8 | 0.048 | 23.4±4.2 |
| Pre-pregnancy BMI classification | | | 0.14 | |
| Normal (18.5 kg/m$^2$ to <25 kg/m$^2$) | 7 (100) | 7 (58) | | 14 (74) |
| Overweight (≥25 kg/m$^2$ to <30 kg/m$^2$) | 0 (0) | 4(33) | | 4 (21) |
| Obese (≥30 kg/m$^2$) | 0 (0) | 1 (8) | | 1 (5) |
| Gestational Weight Gain (kilogram) | 13.6±4.5 | 17.9±4.1 | 0.049 | 16.3±4.6 |
| Gestational Weight Gain (kilogram) during 1st 20 weeks | 4.1±2.2 | 7.6±4.2 | 0.11 | 4.1±2.2 |
| IOM Guidelines | | | 0.05 | |
| Under/Within | 5 (71) | 3 (25) | | 8 (42) |
| Over | 2 (29) | 9 (75) | | 11 (58) |
| Preeclampsia | 1 (14) | 2 (17) | 0.89 | 3 (16) |
| Previous C-section | 0 (0) | 4 (33) | 0.09 | 4 (21) |
| Cesarean Section | 4 (57) | 8 (67) | 0.68 | 12 (63) |
| Pre-term delivery (prior to 37 weeks' gestation) | 1 (14) | 3 (25) | 0.58 | 4 (21) |
| **Maternal Glucose Management** | | | | |
| Age at diagnosis of Insulin-Dependent Diabetes (years) | 17.1±6.9 | 15.1±4.9 | 0.46 | 15.8±5.6 |
| White classification | | | 0.36 | |
| B | 4 (57) | 2 (17) | | 6 (32) |
| C | 1 (14) | 5 (42) | | 6 (32) |
| D | 1 (14) | 3 (25) | | 4 (21) |
| R (Retinopathy) | 0 (0) | 1 (8) | | 1 (5) |
| RF (Retinopathy/Nephropathy) | 1 (14) | 1 (8) | | 2 (11) |
| Mean Glycohemoglobin $A_1$ ($HbA_1$) | | | | |
| First trimester HbA1 (mmol/mol) | 61.0 | 100.0 | 0.01 | 86.0 |
| (%) | 7.7±1.5 | 11.3±2.3 | | 10.0±2.7 |
| Second trimester HbA1 (mmol/mol) | 56.0 | 72.0 | 0.04 | 65.0 |
| (%) | 7.3±1.2 | 8.7±1.4 | | 8.1±1.4 |
| Third trimester HbA1 (mmol/mol) | 52.0 | 64.0 | 0.02 | 60.0 |
| (%) | 6.9±0.59 | 8.0±1.4 | | 7.6±1.3 |
| Mean preprandial glucose over gestation [a] | 132.0±77.1 | 131.1±37.7 | 0.98 | 131.4±49.7 |
| Mean postprandial glucose over gestation [a] | 164.8±37.5 | 166.8±41.9 | 0.92 | 166.1±39.3 |
| Mean insulin dose over gestation [b] | 55.6±15.1 | 96.6±39.1 | 0.005 | 81.5±37.7 |
| Mean insulin dose per kilogram weight over gestation [c] | 0.86 ± 0.27 | 1.26 ± 0.45 | 0.046 | 1.1±0.4 |

| | | | | |
|---|---|---|---|---|
| Mean Arterial Pressure (MAP) 1st 20 weeks of gestation | 87.5±10.9 | 83.6±9.0 | 0.45 | 85.1±9.6 |
| **Offspring Characteristics** | | | | |
| Age of offspring at follow-up (years) | 18.9±3.7 | 21.1±2.9 | 0.18 | 20.3±3.3 |
| Sex (male) | 4 (57) | 8 (67) | 0.68 | 12 (63) |
| Birthweight (grams) | 3891±976 | 3755±498 | 0.69 | 3805±688 |
| Large for Gestational Age | 5 (71) | 7 (58) | 0.57 | 12 (63) |
| Gestational Age (weeks) | 38.6±1.4 | 38.2±1.8 | 0.69 | 38.3±1.6 |
| BMI at follow-up (kg/m$^2$) | 21.6±1.4 | 31.0±7.1 | 0.0007 | 27.5±7.3 |
| BMI of offspring at follow-up waist circumference (cm) over NHLBI guidelines (yes) | 0 (0) | 6 (50) | 0.02 | 6 (32) |

[a] Means were calculated from measurements taken in each trimester

[b] Mean insulin dose over gestation was calculated from measurements of insulin dose taken at approximate weekly visits over full gestation, including at entry to delivery

[c] Mean insulin dose per kilogram weight over gestation was calculated by dividing insulin dose taken at approximate weekly measurements over full gestation by participants' weight (in kg) at that visit.

Large for gestational age was defined as infants with a birthweight >90th percentile, according to gestational age, sex and race.

Waist circumference measurements for offspring >88 cm for females and >102 cm for males was defined as exceeding guidelines per NHLBI.

Data are expressed as mean ± standard deviation are shown for all continuous variables and n (%) are shown for categorical variables. *P < 0.05, **P < 0.01, ***P<0.001.

GWG=gestational weight gain; IOM=Institute of Medicine; BMI=body mass index; cm=centimeter; NHLBI=National Heart, Lung and Blood Institute.

Missing values: marital status, n=1 (5); gestational weight gain in 1st 20 weeks, n=4 (21); mean $HbA_1$ in 1st trimester, n=5 (26%); mean $HbA_1$ in 2nd trimester, n=2 (11); mean preprandial glucose over gestation, n=2 (11); mean MAP, n=3 (16).
